# Ambient Fine Particulate Matter, Nitrogen Dioxide, and Preterm Birth in New York City

**DOI:** 10.1289/ehp.1510266

**Published:** 2016-02-05

**Authors:** Sarah Johnson, Jennifer F. Bobb, Kazuhiko Ito, David A. Savitz, Beth Elston, Jessie L.C. Shmool, Francesca Dominici, Zev Ross, Jane E. Clougherty, Thomas Matte

**Affiliations:** 1 New York City Department of Health and Mental Hygiene, New York, New York, USA; 2 Department of Biostatistics, Harvard T.H. Chan School of Public Health, Boston, Massachusetts, USA; 3 Department of Epidemiology, and; 4 Department of Obstetrics and Gynecology, Brown University, Providence, Rhode Island, USA; 5 Department of Occupational and Environmental Health, University of Pittsburgh Graduate School of Public Health, Pittsburgh, Pennsylvania, USA; 6 ZevRoss Spatial Analysis, Ithaca, New York, USA

## Abstract

**Background:**

Recent studies have suggested associations between air pollution and various birth outcomes, but the evidence for preterm birth is mixed.

**Objective:**

We aimed to assess the relationship between air pollution and preterm birth using 2008–2010 New York City (NYC) birth certificates linked to hospital records.

**Methods:**

We analyzed 258,294 singleton births with 22–42 completed weeks gestation to nonsmoking mothers. Exposures to ambient fine particles (PM2.5) and nitrogen dioxide (NO2) during the first, second, and cumulative third trimesters within 300 m of maternal address were estimated using data from the NYC Community Air Survey and regulatory monitors. We estimated the odds ratio (OR) of spontaneous preterm (gestation < 37 weeks) births for the first- and second-trimester exposures in a logistic mixed model, and the third-trimester cumulative exposures in a discrete time survival model, adjusting for maternal characteristics and delivery hospital. Spatial and temporal components of estimated exposures were also separately analyzed.

**Results:**

PM2.5 was not significantly associated with spontaneous preterm birth. NO2 in the second trimester was negatively associated with spontaneous preterm birth in the adjusted model (OR = 0.90; 95% CI: 0.83, 0.97 per 20 ppb). Neither pollutant was significantly associated with spontaneous preterm birth based on adjusted models of temporal exposures, whereas the spatial exposures showed significantly reduced odds ratios (OR = 0.80; 95% CI: 0.67, 0.96 per 10 μg/m3 PM2.5 and 0.88; 95% CI: 0.79, 0.98 per 20 ppb NO2). Without adjustment for hospital, these negative associations were stronger.

**Conclusion:**

Neither PM2.5 nor NO2 was positively associated with spontaneous preterm delivery in NYC. Delivery hospital was an important spatial confounder.

**Citation:**

Johnson S, Bobb JF, Ito K, Savitz DA, Elston B, Shmool JL, Dominici F, Ross Z, Clougherty JE, Matte T. 2016. Ambient fine particulate matter, nitrogen dioxide, and preterm birth in New York City. Environ Health Perspect 124:1283–1290; http://dx.doi.org/10.1289/ehp.1510266

## Introduction

Numerous studies have suggested associations between air pollution and various birth outcomes, including preterm birth and low birth weight (Nieuwenhuijsen et al. 2013; Stieb et al. 2012). Maternal exposure to ambient levels of particulate matter ≤ 10 and ≤ 2.5 μm in diameter (PM_10_ and PM_2.5_), carbon monoxide (CO), and nitrogen dioxide (NO_2_) has been associated with reduced birth weight and increased odds of low birth weight, summarized in studies by Bosetti et al. (2010) and Stieb et al. (2012). The evidence for an association with preterm birth is more mixed, with results varying from null to weakly positive or negative across exposure levels, pregnancy periods, and methodology (Bosetti et al. 2010; Brauer et al. 2008; Chang et al. 2012; Gehring et al. 2011; Hansen et al. 2006; Stieb et al. 2012; Wilhelm and Ritz 2005).

Preterm birth is an important predictor of infant mortality, childhood morbidity, and possibly adult morbidity (Moster et al. 2008; Saigal and Doyle 2008). Analysis of preterm birth presents unique challenges, particularly the potential for health care practice to influence the timing of labor onset or delivery. A medical intervention (i.e., cesarean section before labor onset, medically induced labor, artificial rupture of membranes before labor onset) that results in preterm birth precludes us from knowing whether a pregnancy would have ended in a spontaneous preterm birth or a term birth. On a population level, such interventions can affect the rate of spontaneous preterm birth as well as the rate of total preterm birth. The use of labor induction and pre-labor cesarean section varies widely across New York City (NYC) hospitals (New York State Department of Health 2015). If these patterns co-vary spatially with air pollution levels in hospital catchment areas, then hospital-level rates of medically indicated births may confound the association between residential air pollution exposure and birth outcomes. The size of our study population allowed us to isolate spontaneous preterm birth, thereby limiting the impact that medical intervention may have on our ability to characterize the relationship between air pollution exposure and preterm birth, as well as an opportunity to examine early preterm birth (< 32 weeks gestation) as an outcome.

Woodruff et al. (2009) identified the need for improved characterization of spatial confounding in studies of air pollution and pregnancy outcomes. Studies control for contextual variables related to socioeconomic status (e.g., Parker and Woodruff 2008; Savitz et al. 2014; Yorifuji et al. 2013) and study region, urban–rural status, or teaching hospital status of the birth facility with the assumption that these represent differences in access to or levels of care along with susceptibility (Parker and Woodruff 2008; Trasande et al. 2013). To our knowledge, no studies have examined potential confounding of individual hospital on the association between air pollution exposure and birth outcomes, a gap which this study seeks to fill. Previously, we reported associations between air pollution (PM_2.5_ and NO_2_) and a reduction in term birth weight (Savitz et al. 2014) but little or no association with hypertensive disorders of pregnancy (Savitz et al. 2015). We made use of the New York City Community Air Survey (NYCCAS) data, which provide highly spatially resolved exposure estimates for geocoded birth certificate data. We also linked birth certificate and hospitalization data, which allowed us to distinguish spontaneous and medically indicated preterm birth and consider additional covariates in the analysis. In this analysis, we investigated the association between estimated maternal exposures to PM_2.5_ and NO_2_ and spontaneous preterm birth and medically indicated preterm births, taking into consideration relevant maternal characteristics including the hospital of birth.

## Methods

### Study Population

**Figure 1:**
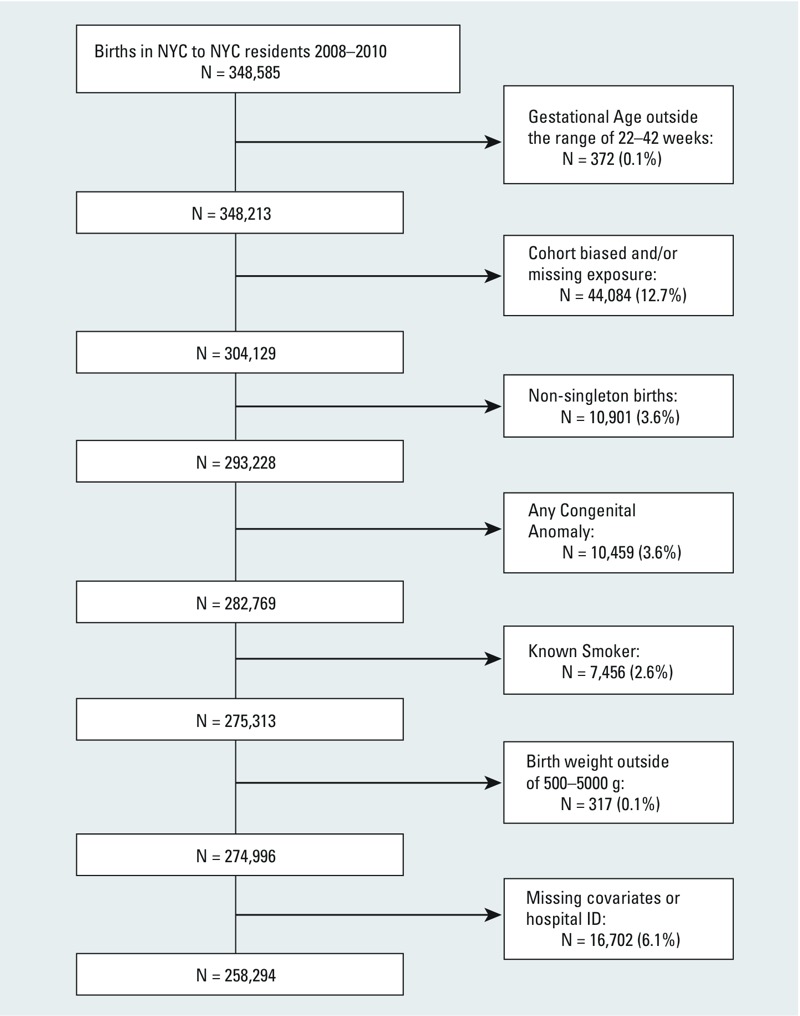
Exclusion process of birth data used in analysis.

We linked records of 348,585 live births to residents of NYC occurring in NYC between 1 January 2008 and 31 December 2010 from the Bureau of Vital Statistics (BVS), New York City Department of Health and Mental Hygiene (NYCDOHMH), to the mothers’ hospitalization records from New York State Department of Health following a study protocol approved by the Institutional Review Board of the BVS NYCDOHMH. Informed consent was not required because this study involved analysis of existing secondary data and posed minimal risk to the subjects. All data were maintained in a password-protected database on a password access–controlled network server at NYCDOHMH with access limited to authorized study investigators. The subset of births used for this analysis was limited to singleton births with 22–42 completed weeks gestation free of congenital malformations to nonsmoking mothers. In addition, we excluded births with an estimated date of conception before 31 July 2007 or after 12 March 2010 (i.e., restricting the conception date range to 22 weeks before the cohort started and 42 weeks before the cohort ended) to avoid the fixed-cohort bias (Strand et al. 2011). Maternal address at the time of delivery was used to locate her residence, but address history during gestation was not available. After further excluding births with missing residence information for assigning exposure, covariate information [maternal age, education, Medicaid status, parity, sex of infant, prepregnancy body mass index (BMI)], hospital of birth, and those with implausible birth weights (< 500 or > 5,000 g), the final analytical data set included 258,294 births (Figure 1).

### Exposure Assignment

The air pollution exposure assignment method for this birth cohort has been described elsewhere (Ross et al. 2013) and used in our analysis of term birth weight (Savitz et al. 2014) and hypertensive disorders of pregnancy (Savitz et al. 2015). Briefly, two sources of air pollution data were combined to estimate exposures to PM_2.5_ and NO_2_ at each mother’s residential address at delivery during gestation: NYCCAS measurements to estimate spatial variation of the pollutants across the city and regulatory monitoring data to adjust for the temporal variation. NYCCAS collected 2-week average concentrations of several pollutants at street level in each of the four seasons, December 2008–December 2010. These values were adjusted for week of monitoring and averaged to create annual averages at the monitoring locations; year 1 data were used to fit land-use regression (LUR) models for each pollutant using buffer-based emission and land-use variables and accounting for residual spatial autocorrelation. Annual average pollutant concentration surfaces for NYC were generated from regression model predictions and averaged within 300-m buffers of each maternal address. These maternal address exposure estimates were then temporally adjusted in 2-week intervals using a citywide time series generated from multiple daily regulatory monitors. Year 2 monitoring data were used for validation of the spatiotemporal estimates; predictions were strongly associated with measured concentrations (*R* ^2^ = 0.83 for PM_2.5_ and 0.79 for NO_2_). We averaged over weeks 1–12 and 13–26 to create first- and second-trimester exposure estimates for each birth and calculated third-trimester cumulative exposures by pregnancy week for use in survival analysis (Chang et al. 2012).

### Birth Outcome and Covariates

**Figure 2:**
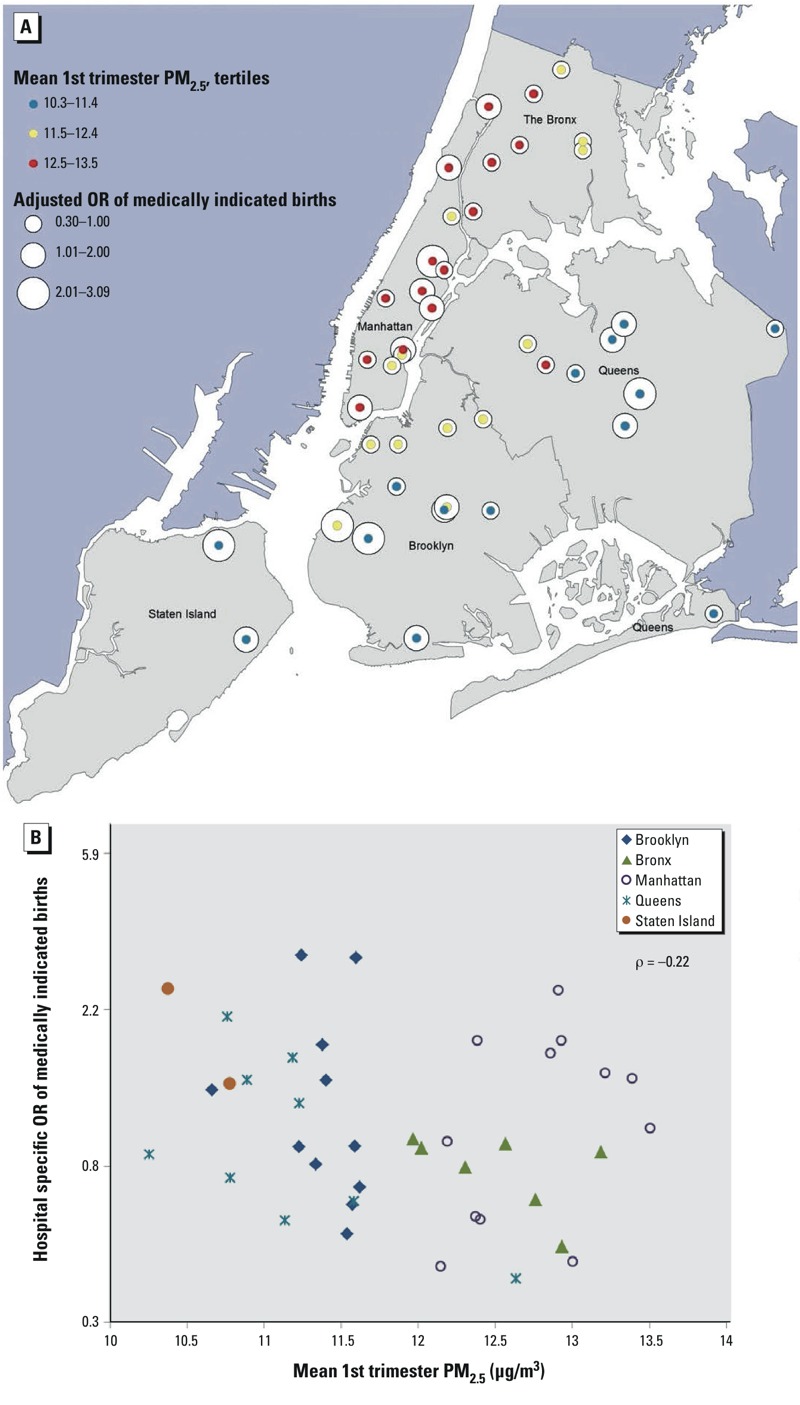
Hospital-specific random intercepts (odds ratios) of medically indicated birth from a logistic mixed model with medically indicated birth compared with spontaneous birth as the outcome, including maternal age, race/ethnicity, education, parity, socioeconomic status, prepregnancy BMI, previous preterm births, and year of birth as covariates, relative to the overall intercept versus hospital mean maternal first-trimester PM_2.5_ exposure (μg/m^3^), by borough, New York City, 2008–2010, map (*A*) and scatter­plot (*B*). Abbreviations: OR, odds ratio; PM_2.5_, particulate matter ≤ 2.5 μm in aerodynamic diameter.

Preterm birth was defined as births with clinical estimate of gestation < 37 completed weeks based on ultrasound; early preterm was defined as < 32 completed weeks. Medically indicated births were identified using either birth or hospital records. A birth with *International Classification of Diseases, 9th Revision* procedure codes for pre-labor artificial rupture of the membrane (73.0, 73.01, 73.09, 73.1, 73.4) or cesarean section performed (74.x) in the absence of a diagnosis of early or threatened labor (644.0x, 644.1x, 644.2x) were considered “medically indicated” as were births recorded in the birth records as having labor induction or cesarean section without trial of labor. We adjusted for covariates known to be associated with preterm birth: maternal age, race/ethnicity stratified by birthplace (non-Hispanic white, U.S./foreign-born non-Hispanic black, U.S./foreign-born Hispanic, U.S./foreign-born Asian, other, and unknown), education (< 9, 9–11, 12, 13–15, 16, or > 16 years), parity (0, 1, 2, ≥ 3), Medicaid status (yes/no), prepregnancy BMI, BMI^2^, and sex of infant. We adjusted for year of conception to control for long-term trends in pollution levels and birth outcomes. As described in our previous analyses (Savitz et al. 2014, 2015), we developed a composite socioeconomic deprivation index (SDI) at the census tract level to address potential confounding by area-level socioeconomic status. Spatial-only maternal exposures to NO_2_ and PM_2.5_ averaged within census tract were negatively correlated with SDI (Pearson correlation coefficients of –0.11 and –0.07, respectively), percent mothers on Medicaid (–0.17, –0.12), and percent without a high school diploma (–0.13, –0.08), and positively correlated with percent mothers with a college degree (0.34, 0.30) and first births to mothers > 35 years of age (0.43, 0.40). In our study population, hospital-level rates of medically indicated birth (not limited to preterm), after adjusting for demographic factors known to influence the likelihood of medically indicated birth (Savitz et al. 2005), were negatively correlated with mean maternal first- and second-trimester PM_2.5_ and NO_2_ exposures (Pearson’s ρ ranging from –0.22 to –0.19). Hospitals with higher rates of medically indicated birth showed a distinct spatial pattern (Figure 2A) and tended to serve mothers with lower residential exposure to PM_2.5_ and NO_2_ (Figure 2B), although adjacent hospitals could have very different rates (e.g. northern Manhattan, Figure 2A). To limit the effect of this spatial confounding, we focused on spontaneous preterm births, incorporated a hospital identifier into our model, and created a variable based on quartiles of hospital-specific rates of medically indicated births, both term and preterm, for hospitals with > 10 births in our data set (≤ 40%; > 40% and ≤ 49%; > 49% and ≤ 62%; and > 62%).

### Analysis

We estimated the association between first- and second-trimester average PM_2.5_ and NO_2_ exposures and preterm birth outcomes using logistic mixed models with random intercepts for hospital of delivery in order to account for residual correlation in the outcome within births at the same hospital after adjusting for covariates. The preterm birth outcomes we examined separately were spontaneous preterm birth, medically indicated preterm birth, spontaneous early preterm birth, and, because of the small numbers of medically indicated early preterm births, all early preterm births. In each case, term birth served as the reference outcome. For each exposure window and outcome, we compared crude and adjusted effect estimates for PM_2.5_ and NO_2_. Adjusted estimates were generated from models that included the individual-level covariates listed above, conception year, SDI, quartiles of medically indicated birth rate, and a random intercept based on hospital identifier. Because our exposure estimation method combined separately estimated spatial exposures with temporal exposures, and our previous analysis of the associations between these two pollutants and birth weight (Savitz et al. 2014) found that the spatial determinant of the exposure contrast contributed more to the observed associations, we estimated the independent effects of the temporal and spatial exposure components on spontaneous preterm birth. To compare across the varying lengths of the third trimester among the births, we used a discrete time survival model with logistic link (Chang et al. 2012) to estimate the effect of cumulative third-trimester spatiotemporal NO_2_ and PM_2.5_ exposures. Due to convergence issues when hospital random intercepts were included in this model specification, we controlled for hospital of delivery using a fixed effect based on hospital identifier (Savitz et al. 2015). We used a 10-μg/m^3^ increase in PM_2.5_ to express odds of preterm birth because this increment is most frequently used in reviews of birth outcome studies (e.g., Sapkota et al. 2012). However, a 10-μg/m^3^ increase in PM_2.5_ is rather a large increase in our data set, representing a change approximately from the 2nd to the 98th percentile. For NO_2_, we used 20 ppb, an increment comparable to the distributional span of the PM_2.5_ increment. Statistical significance of an association was assessed using the alpha level of 0.05.

We conducted additional analyses to assess sensitivity of the results to alternative model specifications. First, we included both PM_2.5_ and NO_2_ in the fully adjusted model. Second, we controlled for ambient temperature by including average temperature in the fully adjusted model for the same exposure time frame. Finally, because our previous analysis of the associations between these two pollutants and birth weight (Savitz et al. 2014) controlled for additional unexplained variation with a random intercept based on neighborhood (census tract), we compared our adjusted model to one with random intercepts based on census tract.

## Results

The distribution of first- and second-trimester exposures in the study population of hospital births to NYC residents 2008–2010 was similar for preterm and term births (Table 1). For PM_2.5_, temporal-only variation was greater than spatial-only variation, whereas for NO_2_, spatial-only variation was greater than temporal-only variation.

**Table 1:** Distribution of first- and second-trimester average PM_2.5_ and NO_2_ overall exposure, temporal-only, and spatial-only exposure for term and preterm births, New York City, 2008–2010.

Exposure	Spontaneous preterm percentiles of exposure					Term percentiles of exposure				
	5th	25th	50th	75th	95th	5th	25th	50th	75th	95th
PM_2.5_ (μg/m^3^)
1st trimester overall	8.7	10.0	11.5	13.3	16.4	8.7	10.1	11.6	13.4	16.7
2nd trimester overall	8.8	10.1	11.4	13.2	16.0	8.8	10.1	11.5	13.3	16.3
1st trimester temporal	8.6	9.5	10.6	12.1	14.6	8.6	9.4	10.6	12.1	14.6
2nd trimester temporal	8.7	9.6	10.6	11.9	14.6	8.7	9.6	10.5	11.9	14.5
Spatial	9.6	10.4	10.9	11.8	13.8	9.7	10.4	11.0	11.9	14.2
NO_2_ (ppb)
1st trimester overall	18.9	23.4	27.1	31.0	37.5	18.8	23.5	27.3	31.5	38.9
2nd trimester overall	18.1	22.5	26.1	30.1	36.9	18.1	22.7	26.4	30.6	38.2
1st trimester temporal	17.6	20.5	22.4	24.7	27.1	17.6	20.4	22.4	24.6	27.1
2nd trimester temporal	17.2	19.3	21.8	23.8	26.9	17.2	19.3	21.8	23.8	26.9
Spatial	19.6	23.5	26.3	28.3	33.2	19.3	23.5	26.5	28.8	35.5
Abbreviations: NO_2_, nitrogen dioxide; PM_2.5_, particulate matter ≤ 2.5 μm in aerodynamic diameter; ppb, parts per billion.										

Demographic characteristics of the study population are shown in Table 2. The preterm birth rate in our study population was 7.4% with 36% of preterm births classified as medically indicated, a proportion consistent with previous reports (Ananth and Vintzileos 2006). The results from the individual-level covariates in the fully adjusted model with overall first-trimester PM_2.5_ exposure (Table 3) indicated that male infants and low or high parity were positively associated with spontaneous preterm birth. Compared with non-Hispanic white mothers, mothers of all other ethnicities had increased odds of spontaneous preterm birth, with highest odds among U.S.-born black and Hispanic mothers. Older mothers had higher odds of spontaneous preterm birth, and mothers with more years of education had lower odds. The odds of spontaneous preterm birth were increased at hospitals in the category with the lowest proportion of medically indicated births, compared with hospitals with the highest proportion [odds ratio (OR) = 1.3; 95% confidence interval (CI): 1.0, 1.7]. Year of conception, BMI, Medicaid status, and SDI were not associated with spontaneous preterm birth.

**Table 2:** Demographic characteristics, preterm birth, and labor type of the study population, New York City, 2008–2010 [*n* (%)].

Characteristic	Total	Preterm (< 37 weeks)		Early preterm (< 32 weeks)
		Spontaneous	Medically indicated	
Infant sex, male	132,654 (51)	6,543 (54)	3,728 (54)	1,536 (53)
Parity
0	120,395 (47)	5,436 (45)	3,273 (48)	1,456 (50)
1	75,976 (29)	3,386 (28)	1,810 (26)	723 (25)
2	35,045 (14)	1,799 (15)	982 (14)	418 (14)
≥ 3	26,878 (10)	1,513 (12)	814 (12)	314 (11)
Maternal ethnicity
Non-Hispanic white	71,085 (28)	1,988 (16)	1,393 (20)	352 (12)
Foreign-born non-Hispanic black	24,999 (10)	1,565 (13)	912 (13)	481 (17)
U.S.-born non-Hispanic black	31,219 (12)	2,399 (20)	1,195 (17)	804 (28)
Foreign-born Hispanic	55,209 (21)	2,493 (21)	1,361 (20)	512 (18)
U.S.-born Hispanic	32,026 (12)	1,870 (15)	992 (14)	442 (15)
Foreign-born Asian	35,227 (14)	1,385 (11)	789 (11)	239 (8)
U.S.-born Asian	3,017 (1)	131 (1)	80 (1)	21 (1)
Other	5,243 (2)	289 (2)	147 (2)	56 (2)
Unknown	269 (0)	14 (0)	10 (0)	4 (0)
Maternal education (years)
< 9	20,712 (8)	1,001 (8)	492 (7)	210 (7)
9–11	45,495 (18)	2,561 (21)	1,354 (20)	645 (22)
12	61,808 (24)	2,998 (25)	1,702 (25)	744 (26)
13–15	56,971 (22)	2,895 (24)	1,738 (25)	795 (27)
16	41,848 (16)	1,621 (13)	921 (13)	334 (11)
> 16	31,460 (12)	1,058 (9)	672 (10)	183 (6)
Maternal age (years)
< 20	17,065 (7)	954 (8)	518 (8)	238 (8)
20 to < 25	53,233 (21)	2,410 (20)	1,371 (20)	613 (21)
25 to < 30	68,101 (26)	2,910 (24)	1,672 (24)	663 (23)
30 to < 35	68,376 (26)	3,044 (25)	1,757 (26)	714 (25)
35 to < 40	39,918 (15)	2,044 (17)	1,164 (17)	514 (18)
≥ 40	11,601 (4)	772 (6)	397 (6)	169 (6)
Mother on Medicaid	157,629 (61)	7,773 (64)	4,344 (63)	1,892 (65)
Year of conception
2007	43,205 (17)	2,065 (17)	1,153 (17)	455 (16)
2008	99,569 (39)	4,749 (39)	2,675 (39)	1,181 (41)
2009	96,124 (37)	4,419 (36)	2,536 (37)	1,052 (36)
2010	19,396 (8)	901 (7)	515 (7)	223 (8)
Maternal prepregnancy body mass index
< 18.5	14,264 (6)	655 (5)	356 (5)	129 (4)
18.5 to < 25	139,480 (54)	5,878 (48)	3,223 (47)	1,234 (42)
25 to < 30	61,388 (24)	3,075 (25)	1,735 (25)	818 (28)
≥ 30	43,162 (17)	2,526 (21)	1,565 (23)	730 (25)
Total births	258,294 (100)	12,134 (100)	6,879 (100)	2,911 (100)

**Table 3:** Odds ratios of spontaneous preterm birth for covariates in fully adjusted model.*^a^*

Covariate	OR (95% CI)
Mother’s ethnicity/birthplace
Non-Hispanic white	1.0
Foreign-born black	1.7 (1.6, 1.8)
U.S.-born black	2.3 (2.2, 2.5)
Foreign-born Hispanic	1.4 (1.3, 1.5)
U.S.-born Hispanic	2.0 (1.8, 2.1)
Foreign-born Asian	1.4 (1.3, 1.5)
U.S.-born Asian	1.7 (1.4, 2.0)
Other	1.9 (1.7, 2.1)
Unknown	1.9 (1.1, 3.2)
Mother’s age (years)
< 20	1.0 (0.9, 1.1)
20 to < 25	1.0
25 to < 30	1.1 (1.0, 1.2)
30 to < 35	1.3 (1.2, 1.4)
35 to < 40	1.6 (1.5, 1.7)
≥ 40	2.1 (1.9, 2.3)
Mother’s education (years)
< 9	0.9 (0.8, 1.0)
9–11	1.0
12	0.9 (0.9, 1.0)
13–15	0.9 (0.8, 0.9)
16	0.8 (0.7, 0.8)
> 16	0.7 (0.7, 0.8)
Number of previous live births
None	1.1 (1.1, 1.2)
1	1.0
2	1.0 (1.0, 1.1)
≥ 3	1.1 (1.1, 1.2)
Medicaid status
Yes	0.9 (0.9, 1.0)
No	1.0
Conception year
2007	1.0 (1.0, 1.1)
2008	1.0 (0.9, 1.1)
2009	1.0 (0.9, 1.1)
2010	1.0
SDI score	1.0 (1.0, 1.1)
Sex of infant
Male	1.1 (1.1, 1.2)
Female	1.0
Prepregnancy body mass index
Linear	1.0 (1.0, 1.0)
Quadratic	1.0 (1.0, 1.0)
Hospital rate of medically indicated births
≤ 40%	1.3 (1.0, 1.7)
> 40 and < 49%	1.2 (0.9, 1.7)
≥ 49% and ≤ 62%	1.1 (0.8, 1.5)
> 62%	1.0
^***a***^From model including first-trimester PM_2.5_ and a hospital identifier random intercept.	

**Figure 3:**
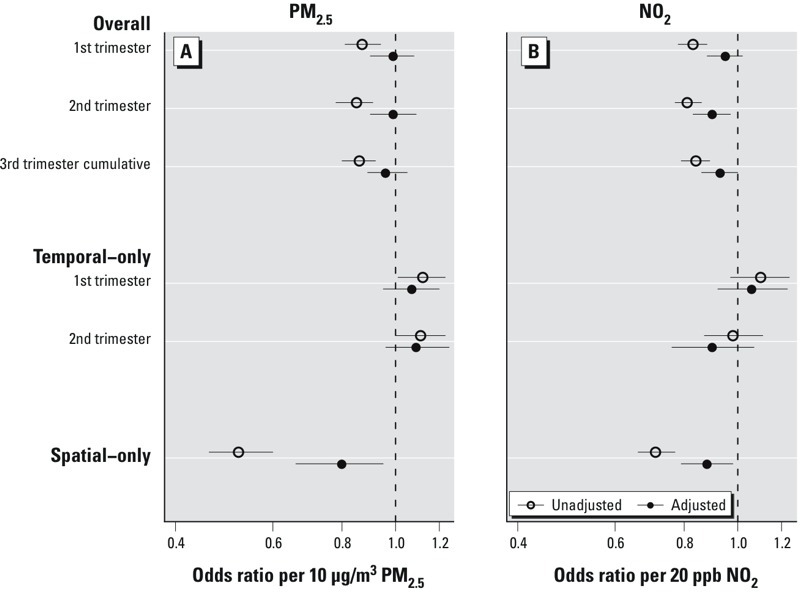
Odds ratios and 95% confidence intervals of spontaneous preterm birth for overall, temporal-only, and spatial-only exposures: (*A*) per 10 μg/m^3^ PM_2.5_; (*B*) per 20 ppb of NO_2_ exposure. For first- and second-trimester average exposures, the adjusted logistic mixed model included individual demographics (mother’s ethnicity/birthplace, age, education, parity, Medicaid status, prepregnancy BMI, sex of infant, conception year), SDI, hospital rates of medically indicated births and random intercept by hospital ID. For third-trimester cumulative exposures, the discrete time survival analysis was adjusted for individual demographics, SDI, and fixed effect of hospital.

Higher overall second-trimester NO_2_ exposure was negatively associated with spontaneous preterm birth in the fully adjusted model (OR = 0.90; 95% CI: 0.83, 0.97 per 20 ppb); there was no association between overall average first-, average second-, or cumulative third-trimester PM_2.5_ exposure and preterm birth (Figure 3). In the unadjusted models, both pollutants were significantly negatively associated with spontaneous preterm and early preterm birth across the overall exposures. The temporal contribution to PM_2.5_ exposure was positively associated with spontaneous preterm birth (OR = 1.2; 95% CI: 1.01, 1.23 per 10 μg/m^3^ in the crude model to 1.07; 95% CI: 0.95, 1.21 in the adjusted model), whereas NO_2_ was not significantly associated with spontaneous preterm birth in either the adjusted or unadjusted models. For the spatial-only exposures, both PM_2.5_ and NO_2_ were significantly negatively associated with spontaneous preterm birth, adjusted or unadjusted.

**Figure 4:**
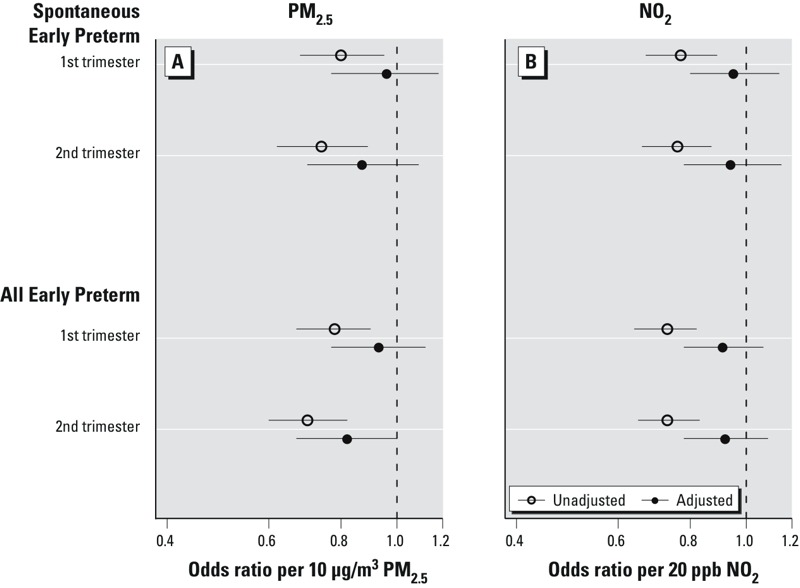
Odds ratios and 95% confidence intervals of spontaneous-only and combined spontaneous and medically indicated early preterm births (< 32 weeks) per 10 μg/m^3^ of overall first- and second-trimester exposure to PM_2.5_ (*A*) and 20 ppb of NO_2_ (*B*). The adjusted model included individual demographics (mother’s ethnicity/birthplace, age, education, parity, Medicaid status, prepregnancy BMI, sex of infant, conception year), SDI, hospital rates of medically indicated births and random intercept by hospital ID.

There was no evidence of association between either pollutant and early preterm birth in the adjusted models (Figure 4). Medically indicated and all preterm birth followed the same pattern of associations with the pollutants as spontaneous preterm birth (Table 4).

**Table 4:** Comparison of spontaneous preterm birth odds ratio (95% CI) with medically indicated preterm birth and all preterm birth odds ratio (95% CI) for PM_2.5_ (per 10 μg/m^3^) and NO_2_ (per 20 ppb) using estimated overall exposures in unadjusted and adjusted models.

Pollutant	Exposure trimester	Model	Spontaneous preterm births	Medically indicated preterm births	All preterm births
PM_2.5_	1st	Crude	0.87 (0.81, 0.94)	0.81 (0.73, 0.89)	0.85 (0.80, 0.90)
	1st	Fully adjusted	0.99 (0.90, 1.08)	0.94 (0.84, 1.06)	0.97 (0.90,1.05)
	2nd	Crude	0.85 (0.78, 0.91)	0.80 (0.73, 0.89)	0.83 (0.78, 0.88)
	2nd	Fully adjusted	0.99 (0.90, 1.09)	0.95 (0.84, 1.08)	0.95 (0.88, 1.03)
NO_2_	1st	Crude	0.83 (0.78, 0.88)	0.77 (0.71, 0.84)	0.81 (0.77, 0.85)
	1st	Fully adjusted	0.94 (0.87, 1.02)	0.90 (0.81, 0.99)	0.92 (0.87, 0.98)
	2nd	Crude	0.81 (0.77, 0.86)	0.78 (0.72, 0.84)	0.80 (0.76, 0.84)
	2nd	Fully adjusted	0.90 (0.83, 0.97)	0.89 (0.80, 0.99)	0.89 (0.83, 0.95)
Abbreviations: PM_2.5_, particulate matter ≤ 2.5 μm in aerodynamic diameter; μg/m^3^, micrograms per cubic meter; NO_2_, nitrogen dioxide; ppb, parts per billion. All term births serve as the reference outcome.					

**Figure 5:**
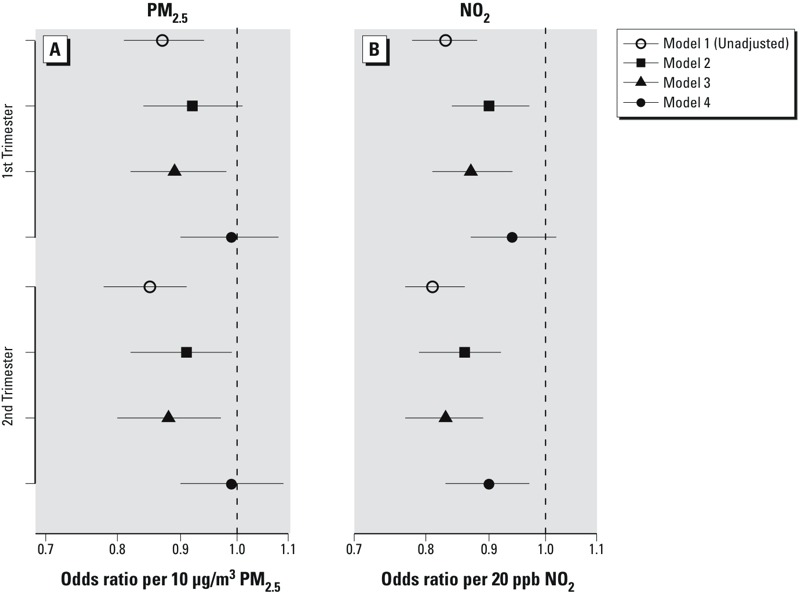
Odds ratios and 95% confidence intervals of spontaneous preterm birth per 10 μg/m^3^ estimated overall first- and second-trimester PM_2.5_ (*A*) and 20 ppb NO_2_ (*B*) exposure based on single-pollutant models with the following different degrees of confounder adjustment: model 1: unadjusted; model 2: adjustment with individual demographics (mother’s ethnicity/birthplace, age, education, parity, Medicaid status, prepregnancy BMI, sex of infant, conception year), SDI and random intercept by census tract; model 3: adjustment with individual demographics, SDI, random intercept by census tract, plus quartile of hospital rates of medically indicated births; model 4: adjustment with individual demographics, SDI, hospital rates of medically indicated births and random intercept by hospital ID (fully adjusted model).

When we adjusted the pollutants for one another in the fully adjusted model with overall exposures, the odds ratios for PM_2.5_ moved slightly above the null (OR = 1.03; 95% CI: 0.92, 1.14 and 1.06; 95% CI: 0.95, 1.18 for first- and second-trimester exposures, respectively), and those for NO_2_ became slightly more negative (OR = 0.93; 95% CI: 0.85, 1.02 and 0.87; 95% CI: 0.80, 0.96, respectively). Including ambient temperature in various alternative models had little impact on the estimates; second-trimester PM_2.5_ OR in the fully adjusted model with temperature was 0.97 (95% CI: 0.88, 1.07) compared with 0.99 (95% CI: 0.90, 1.09) without, similar to first-trimester NO_2_ (OR = 0.92; 95% CI: 0.84, 1.00 and 0.94; 95% CI: 0.87, 1.02 for with and without temperature, respectively). In the analysis to evaluate alternative spatial random intercepts (Figure 5), we found that for both PM_2.5_ and NO_2_, including a random intercept based on census tract in a model with covariate adjustment but no hospital level information (model 2) moved the apparent negative crude association (model 1) slightly closer to the null. The addition to model 2 of a four-level variable based on quartiles of hospital rates of medically indicated births (model 3) shifted the pollutant effect estimates closer to the unadjusted estimates. The substitution of hospital identifier for census tract in the random intercept (model 4, or the fully adjusted model, presented in Figure 2) substantively attenuated the negative associations for both pollutants and reduced the PM_2.5_ estimates to null.

## Discussion

In this study, we did not find evidence that either PM_2.5_ or NO_2_ exposure was positively associated with spontaneous preterm delivery in NYC. Rather, we found modest evidence of inverse (“protective”) associations between these pollutants and preterm birth in the unadjusted models. Adjusting for individual maternal characteristics, census tract–level social deprivation, random intercepts for either census tracts or hospitals, and the level of medically indicated birth rate at hospitals moved these negative associations for both pollutants closer to the null. The odds ratios for PM_2.5_ became null, but the associations for NO_2_ remained negative and statistically significant for the second trimester. By analyzing the spatial-only, temporal-only, and overall exposures, we found that the negative associations were mainly driven by the spatial exposure variation, originating from the common spatial pattern of air pollution and delivery hospitals in which hospitals with higher average exposures among their patients tended to have lower rates of spontaneous preterm birth (Figure 2). Because *a*) a negative association between spontaneous preterm birth and air pollution is not biologically plausible, *b*) the hospital-level rates of medically indicated birth were negatively correlated with maternal air pollution exposures (which is unlikely to be a causal relationship), and *c*) the pattern of negative associations with the pollutants are very similar for spontaneous preterm births and medically indicated preterm births, the moderate negative association between NO_2_ and spontaneous preterm birth in the fully adjusted model is a consequence of residual confounding.

A recent systematic review of air pollution, birth weight and preterm birth by Stieb et al. (2012) found that although most of the studies reported reduced birth weight and increased odds of low birth weight in relation to air pollutants including PM_2.5_ and NO_2_, fewer effect estimates were available for preterm births studies and that their results were more mixed. Some of the estimates were significantly negative for NO_2_ (Jalaludin et al. 2007) and PM_2.5_ (Wilhelm and Ritz 2005). The variation in study design, exposure assignment method, geographic scale, and length of study period across these studies may explain some of the inconsistency in their findings, but preterm birth itself likely presents additional methodologic complexity in determining its relationship with air pollution, more so than analysis of birth weight. It is well known that hospital characteristics and provider characteristics can influence the rate of medically indicated birth (Clark et al. 1998; Goyert et al. 1989), and increased frequency of labor induction may account for the increasing rate of preterm birth seen nationwide in the United States (MacDorman et al. 2002). The results from our study may be influenced by the increased spatial resolution of the exposure assessment resulting from the use of a high-density monitoring network. However, given that variation among hospitals can occur at any spatial scale (e.g., among hospitals within a state or multi-state region) and may be influencing our ability to detect pollution effects on preterm birth, the nature of the confounding we observed in this analysis is unlikely to be limited to our study setting and spatial resolution.

A preterm birth can be spontaneous or medically indicated, and one would expect that the impact of air pollution, if it exists, would be more clearly seen on spontaneous preterm birth than on medically indicated birth, although the censoring of potential future spontaneous preterm births through medical intervention may also affect the observed frequency of spontaneous preterm births. Only two past studies that examined air pollution and preterm birth analyzed spontaneous and medically indicated preterm birth separately: One study (Darrow et al. 2009), a time-series design, found essentially null associations with multiple pollutants even when induced preterm birth was excluded; the other study (Lee et al. 2012) found a somewhat stronger PM_2.5_ association with induced/indicated preterm than with spontaneous preterm birth, though not significantly stronger. The relatively large sample size of our data set allowed us to focus on spontaneous preterm birth separately from medically indicated preterm birth. The pattern of association with the air pollutants was similar, although consistently more negative for medically induced preterm birth, potentially caused by hospital/patient characteristics that increase the use of medical intervention in parts of the city that coincidentally have lower pollution levels. Although we observed some spatial clustering of hospitals with high or low rates of medically indicated birth, we also observed that two adjacent hospitals can have very different rates (data not shown). Including a measure of the hospital-level rate of medically indicated labor in our analysis did not attenuate the negative association between air pollution and preterm birth as much as including random intercepts for delivery hospital, suggesting that other provider or patient characteristics that vary among hospitals likely confound the association between air pollution and preterm births, rather than the tendency for medically indicated birth only. To our knowledge, none of the past studies of air pollution and preterm birth have considered potential spatial confounding by hospital of birth.

Our exposure estimation method was conducive to separately estimating odds ratios for temporal-only and spatial-only exposures. We found that the negative associations were largely driven by the spatial exposure and those associations were more affected by covariate adjustment. Because maternal characteristics of NYC residents are unlikely to have substantively changed in the 3-year study period, the weak influence of covariate adjustment on the temporal-only association was expected. The attenuation of the strong negative association between spatial exposure of NO_2_ and PM_2.5_ and preterm birth toward the null suggests that refinements on the control of spatial confounding, beyond neighborhood of residence and birth hospital, may help to clarify the underlying nature of associations between these pollutants and preterm birth. Past studies of air pollution and preterm birth, except for Wu et al. (2011), did not examine temporal and spatial exposures separately, and often it is ambiguous whether an association is driven by the spatial or temporal component of estimated exposures. The Wu et al. (2011) study considered alternative methods to estimate air pollution exposures and found positive associations for preterm and early preterm birth with higher levels of NO_2_ in Los Angeles, California, as estimated from temporally adjusted LUR model exposure estimates, but noted that the associations were stronger when estimates were unadjusted for temporal variation. In our analysis, temporal-only exposure estimates showed null associations for PM_2.5_ and first-trimester NO_2_, consistent with Darrow et al.’s (2009) time-series analysis of preterm birth and air pollution; however, the significantly negative second-trimester association seen for the overall NO_2_ exposure in our study also is seen in the temporal-only component and deviates from Darrow et al.’s findings. Future studies, especially those using LUR-based exposure estimation, may benefit from analyzing spatial and temporal exposure estimates separately to identify whether the association (or confounding) comes from spatial or temporal components of exposure.

Limitations of our study include potential exposure misclassification error due to estimates only at mother’s residence at delivery, without details on maternal residential mobility during pregnancy, place and type of work, variation in indoor/outdoor activity, or building characteristics, including floor of residence. The fact that a substantive fraction of the mothers in this study were foreign born leaves some uncertainty regarding the influence of spatial variation of air pollution on the baseline health of these mothers. We were also unable to address all known confounders, including noise and environmental tobacco smoke.

## Conclusions

Overall, our results do not provide evidence supporting an association between exposure to PM_2.5_ or NO_2_ and increased numbers of preterm births. The negative association we observed appears to have been confounded by hospital characteristics, which covary with pollutant levels of the patients seen at different hospitals in New York City.
